# From stress to exhaustion: the mediating role of meaning of work in the relationship between role conflict and emotional exhaustion among preschool teachers

**DOI:** 10.3389/fpsyg.2026.1828845

**Published:** 2026-07-15

**Authors:** Lisu She, Hongyu Hu

**Affiliations:** 1 Office of Foreign Affairs and Cooperation, Sichuan Preschool Educators College, Mianyang, Sichuan, China; 2 College of Education, Mianyang Teachers’ College, Mianyang, Sichuan, China

**Keywords:** emotional exhaustion, meaning of work, mediating effect, preschool teachers, role conflict

## Abstract

**Background:**

Emotional exhaustion is common among preschool teachers and is associated with poorer well-being and teaching quality. Previous studies have mainly focused on individual stressors such as workload, while paying less attention to role conflict and work-related resources such as meaning of work. Using the Job Demands-Resources model as a framework, this research looked into how work meaning connects with role conflict and emotional exhaustion.

**Methods:**

This study collected data from 486 preschool teachers from three Chinese provinces through a questionnaire survey. The measures included role conflict, meaning of work, and emotional exhaustion, and the proposed mediation model was tested.

**Results:**

Role conflict is positively correlated with emotional exhaustion (*β* = 0.594, *p* < 0.001). Meaning of work partially mediates this relationship, explaining 35.19% of the total effect (*β* = 0.208, *95% CI* [0.142, 0.274]). Meaning of work varies according to age (*p* = 0.005) and teaching experience (*p* = 0.003). Teachers aged 20–30 and those with 4–10 years of experience reported higher levels of meaning of work. No significant group differences in emotional exhaustion were found across the examined demographic variables.

**Conclusion:**

In the results, for preschool teachers, meaning of work plays a partial mediating role between role conflict and emotional exhaustion. These results emphasize how crucial it is to create a meaningful workplace in order to promote teachers’ wellbeing.

## 1 Introduction

Preschool teachers are a distinct group, managing multiple responsibilities such as teaching, providing care, communicating with families, and handling administrative duties (Kurniati and Nibrosurrahman, 2025). Unlike teachers in other stages, kindergarten teachers handle both instruction and full-time care of young children, while also communicating frequently with parents (Li et al., 2025). The overlap of these varied role expectations frequently involves goal conflicts and heightened stress levels (Aboagye et al., 2024). Long-term exposure to this pressure will lead to emotional exhaustion (Bakker and de Vries, 2021), which is characterized by fatigue, decreased enthusiasm for work and increasing alienation from responsibility (Afota et al., 2024). Research shows that Chinese pre-school teachers have general occupational burnout, and the overall burnout rate is as high as 53.2%, of which 38.6% of teachers experience emotional exhaustion (Li et al., 2020). Emotional exhaustion in teachers is linked to lower job satisfaction and dedication, higher teacher mobility, and negative outcomes for the quality and sustainability of early childhood education (Klusmann et al., 2022). Resolving the issue of emotional exhaustion is essential to maintaining the stability and professionalism of the teaching staff as early childhood education standards continue to improve (Pan et al., 2026). Understanding the psychological drivers of emotional exhaustion, along with where meaning of work fits into this process, matters for creating good support systems and reducing professional burnout.

Through the job demand-resources (JD-R) model (Demerouti et al., 2001), the pressure and adaptability of preschool teachers can be effectively understood. The model explains the results of career development by linking the decline in health and work motivation with the balance of job needs and work resources. Within this framework, role conflicts count as a common work demand, while meaning of work serves as a key work resource. Both of these factors are related to emotional exhaustion, which is a health-related outcome. Role conflict is the core work need in the preschool education environment and has been widely studied (Dehne et al., 2025; Pretorius and Padmanabhanunni, 2025). Role conflicts occur when teachers face conflicting work expectations, such as balancing the roles of educators and caregivers, or achieving a balance between professional autonomy and compliance with administrative requirements (Huang et al., 2024). This issue is common in preschool education and is linked to higher levels of burnout, dissatisfaction, and turnover intentions (Aboagye et al., 2025; Chakravorty and Singh, 2021). Psychological resources, such as meaning of work, resilience, and self-efficacy, affect teachers’ vulnerability to emotional exhaustion (Bao, 2024; Sun and Yin, 2025). Meaning of work, which is a profound inherent resource and a mediator between the interaction between emotional exhaustion and outside sources of pressure, can directly contribute to teachers’ enthusiasm for careers (Suyatno et al., 2021). A high meaning of work helps teachers get psychological compensation from daily work and delay emotional exhaustion; while the lack of meaning of work may increase the risk of professional burnout (Trillo et al., 2024).

A large body of research has looked into how meaning of work acts as a moderator (Chakravorty et al., 2025; Schadenhofer et al., 2018). However, the mediating pathway through which role conflict may relate to emotional exhaustion by reducing this core psychological resource remains not fully studied. This research, grounded in the JD-R model, explores whether meaning of work serves as a mediator in the link between role conflicts and emotional exhaustion among Chinese early childhood teachers. The research results will clarify the psychological relationship between work pressure and emotional exhaustion, and provide theoretical basis and practical suggestions for preventing professional burnout in early childhood teachers.

## 2 Theory and hypotheses

### 2.1 Theoretical framework

This study adopts the JD-R model from Demerouti et al. (2001) as its core theoretical foundation. Within the JD-R model, every aspect of the workplace splits into two broad types: job demands and job resources. Job demands are defined as job elements needing ongoing physical and mental exertion. Job resources, in contrast, refer to the psychological, social, and organizational features that assist people in handling work challenges and fostering professional development. These two dimensions underpin the health impairment and motivational processes, respectively, which are used to explain the differentiation in employees’ occupational well-being. In this research, emotional exhaustion serves as a key sign of health decline, with role conflict and meaning of work seen as an important work demand and a work resource, respectively. Preschool teachers must balance multiple roles, including educators, caregivers, administrators, and communication bridges between families and schools. The conflicts and contradictions between these different role expectations will cause continuous psychological pressure, which is consistent with the characteristics of work needs that require continuous investment in physical and mental resources (Seo et al., 2023). Meaning of work arises from a teacher’s internalization of personal professional values and sense of professional identity. It provides continuous psychological energy and emotional support, thus fitting the basic functions of work resources and helping individuals cope with challenges and achieve growth (Choi et al., 2025). Emotional exhaustion is the result of long-term consumption of emotional resources. It represents the loss of an individual’s ability to restore balance between giving and recovery, making it a typical result of the path of health deterioration (Afota et al., 2024). This study incorporates role conflict, meaning of work, and emotional exhaustion within the job-demand, job-resource, and health-impairment pathways of the JD-R model. It proposes a mediation framework to clarify how role conflict may exacerbate emotional exhaustion by reducing meaning of work.

### 2.2 Role conflict and emotional exhaustion

Role conflict refers to individuals facing conflicting needs at work (Thi et al., 2025). For preschool teachers, role conflicts usually manifest in three main forms: the contradiction between the dual responsibilities of educators and carers, the challenge of balancing career autonomy and administrative expectations, and the difference between family and school expectations (Seo et al., 2023). The JD-R model states that these competing role needs reflect the professional demands that necessitate the deployment of emotional and cognitive resources by educators. Studies show that role conflict and feelings of emotional exhaustion go together strongly in a positive direction (Asfahani, 2022; López-Cabarcos et al., 2023; Xu, 2019). For example, Aboagye et al. (2024) found that among preschool instructors in low- and middle-income nations, emotional exhaustion and high job needs (including role conflicts) were closely related. In addition, the research of Li et al. (2023) demonstrated that the cognitive and job requirements of preschool teachers were positively correlated with emotional exhaustion. The study found a connection from role conflict to turnover intention, where emotional exhaustion served as a partial mediator. The role conflict of preschool teachers usually stems from the inconsistency between educational concepts and practices, the uneven distribution of working hours, and the gap between professional responsibilities and social expectations (Su and Chung, 2023). Emotional exhaustion results from the persistent depletion of psychological resources caused by these conflicts (Brown et al., 2023). However, in hospital settings, Mwakyusa and McHaro (2024) discovered a non-significantly negative connection between role conflict and emotional exhaustion. While the majority of studies have shown a positive connection between role conflict and emotional exhaustion, mixed findings still exist, particularly in preschool education. Thus, the following hypothesis is put out by this study:

*H1*: Among preschool teachers, role conflict and emotional exhaustion are significantly positively correlated.

### 2.3 Role conflict, meaning of work, and emotional exhaustion

Meaning of work is defined as how much people view their job as important, goal-directed, and matching their own values (Steger et al., 2012). Within the JD-R framework, meaning of work is an important psychological resource. Role conflict may lower preschool teachers’ meaning of work because conflicting expectations can make them feel that their daily work is increasingly disconnected from their professional values and educational goals. When much of their time and energy is spent dealing with incompatible demands, they may gradually perceive their work as less meaningful (Demerouti et al., 2001). For example, Monnot and Beehr (2014) discovered that work role stressors like role conflict and role ambiguity negatively impacted employees’ Meaning of work. Similarly, Geisler et al. (2019) reported a negative link between role conflict and meaning in work among human service workers. Longitudinal evidence has also shown that adverse psychosocial work characteristics and role-related demands can weaken employees’ perceived meaning at work over time (Clausen and Borg, 2011). In the context of Chinese preschool education, Liu and Wong (2026) further noted that teachers often experience identity tensions between performative institutional demands and their commitment to caring for and supporting children’s development, which may further diminish their sense of work meaning. Given the JD-R model and existing empirical findings, a negative association is expected between role conflict and preschool teachers’ meaning of work.

**Figure 1:**
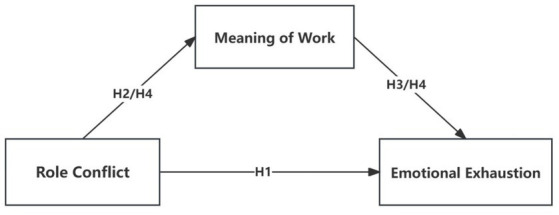
Hypothesized model.

In addition, meaning of work and emotional exhaustion show a strong correlation with each other (Guidetti et al., 2021; Polatcan et al., 2025). For example, Trillo et al. (2024) found that in Spain, emotional exhaustion and meaning of work were strongly and inversely related. Hong et al. (2025) also verified this negative association within a Chinese context, with meaning of work serving as a mediator between organizational support and emotional exhaustion. Furthermore, research examining organizational dehumanization revealed that the violation of work purpose fully mediates the link between dehumanization and emotional exhaustion (Cheung, 2025). This supports the notion that stress lowers the perceived meaningfulness of work, bringing about emotional exhaustion. According to the JD-R model, role conflict reduces the professional significance that instructors gain from their work, with emotional exhaustion as a possible outcome. More specifically, role conflicts required by work may reduce meaning of work, thus aggravating emotional exhaustion. A central psychological path from role conflict to emotional exhaustion runs through meaning of work. In light of this, we put out the following theories (Figure 1):

*H2*: Among preschool teachers, role conflict has a negative correlation with meaning of work.

*H3*: Preschool instructors' emotional exhaustion is inversely correlated with meaning of work.

*H4*: Meaning of work serves as a mediator between role conflict and emotional exhaustion.

## 3 Methods

### 3.1 Sample and data collection procedures

Data for this study were collected between October and November 2025 from kindergartens located in three Chinese provinces: Hainan, Hubei, Guangdong, representing southern and central regions of China. Through an online platform, teachers filled out the survey, and all procedures followed the ethical standards set by the Declaration of Helsinki. The selection of kindergartens followed these inclusion criteria: (a) they were formally registered early childhood education institutions; (b) they employed at least 10 full-time teachers; (c) they were located in urban or suburban areas; and (d) they agreed to participate voluntarily. Kindergartens that were exclusively special education institutions or had been operating for less than 1 year were excluded. Based on an estimated 20% attrition rate (Kline, 2018), a minimum of 408 participants was required. All 520 of the surveys that were sent out were returned. 486 legitimate surveys were included after careless or incomplete responses were eliminated, yielding a 93.5% response rate. With such a high response rate (93.5%), the potential impact of non-response bias on our statistical results is minimal, as the proportion of non-respondents (6.5%) is well below the commonly accepted threshold for serious bias (Rogelberg et al., 2007). The final sample’s demographic characteristics are shown in Table 1. The sample reflects the current gender distribution of pre-school education practitioners in China, including 52 male teachers (10.7%) and 434 female teachers (89.3%). Recent studies emphasize the problem of gender imbalance, pointing out that male teachers face challenges such as shortage, high mobility and marginalization (Wang et al., 2024; Zhang et al., 2022). Given this substantial gender imbalance, any interpretation related to gender should be made with caution. Although the sample covers multiple provinces, it does not represent all regions of China, and regional variation should be examined more fully in future research.

**Table 1:** Participants’ demographic details.

Constructs	Category	Frequency	Percentage (%)
Gender	Male	52	10.7
	Female	434	89.3
Age	20–30 years	186	38.3
	31–40 years	212	43.6
	41–50 years	57	11.7
	Above 51	31	6.4
Teaching experience	1–3 years	63	13.0
	4–10 years	178	36.6
	Above 11	245	50.4
Job position	Lead teacher	264	54.3
	Assistant teacher	222	45.7
Employment status	Permanent teacher	268	55.1
	Non-permanent teacher	218	44.9
Type of kindergarten	Public	370	76.1
	Private	116	23.9
Highest education level	Associate degree or below	431	88.7
	Bachelor’s degree or above	55	11.3

### 3.2 Measures

#### 3.2.1 Role conflict

To assess role conflict among pre-school teachers in the workplace, this study adopts the role conflict scale created by Rizzo et al. (1970). The scale contains 8 entries (for example, “I have to do something I do not want to do” and “I have to deal with some unnecessary work”), which requires teachers to score their own experiences. The scale adopts the 7-point Likert scale, of which 1 means “very disagree” and 7 means “very agree.” The scale has good internal consistency and has been verified in China (Zhou et al., 2023). In this study, the Cronbach’s *α* was 0.866.

#### 3.2.2 Meaning of work

The Work Meaning Scale from Steger et al. (2012) is used in this study to assess how preschool teachers perceive meaning of work. In this study, meaning of work is defined as teachers’ perceived importance, purpose, and value in their present job. This scale consists of 10 items (e.g., “my work has a higher goal and meaning”), using a 7-point Likert scale where 1 stands for “strongly disagree” and 7 stands for “strongly agree.” One item (“my work has no impact on the world”) was reverse-coded. Following reverse coding of the negatively worded item, the mean score was computed, where a higher value reflected a greater degree of work meaningfulness. Although this scale has a multidimensional structure, the present study uses the overall score as a composite indicator of meaning of work, because our hypotheses focus on preschool teachers’ general level of meaning of work rather than on differences among specific dimensions. This method also matches earlier research that used the scale as an overall measure of work meaning. The scale has shown good validity and reliability in many Chinese studies (Hong et al., 2025). The Cronbach’s *α* was 0.903 in this study.

#### 3.2.3 Emotional exhaustion

This study uses the emotional exhaustion scale in the Maslach Occupational Burnout Scale (MBI) developed by Maslach et al. (1997) to measure the emotional exhaustion of preschool teachers. The scale contains 9 entries (for example, “my work exhausts my energy”), using the 7-point Likert scale, 1 means “very disagree” and 7 means “very much agree.” The scale has good internal consistency and has been verified in China (Zhang et al., 2022). For this study, Cronbach’s α came out to 0.883.

### 3.3 Statistical analysis

SPSS 26.0 was used to analyze the data for this investigation. First, scale validity and reliability were examined, with descriptive statistics coming next. The mediating effects were tested using Model 4 in the SPSS PROCESS 4.2 macro (Hayes, 2022) and through group difference analysis. Gender, age, teaching experience, and job position were included as control variables in the mediation analysis because these demographic characteristics may be related to preschool teachers’ work experiences and the focal study variables. To test the mediation effect, percentile bootstrap confidence intervals were generated using 5,000 bootstrap resamples (Efron, 1992). The absence of zero within the 95% confidence interval signified that the indirect effect attained statistical significance (Hayes and Rockwood, 2017).

## 4 Results

### 4.1 Preliminary analyses

#### 4.1.1 Common method bias (CMB)

The first component explained 38.526% of the total variance, below the 40% cutoff (Podsakoff et al., 2003). In addition, bias was further assessed through the Common Latent Factor (CLF) approach. The difference between the baseline model (*χ*^2^ = 416.091, df = 321) and the model with the latent factor (*χ*^2^ = 418.072, df = 322) was 1.981 (*p* > 0.05), which was not statistically significant (Podsakoff et al., 2003). This suggests that CMB is not a serious problem. Nevertheless, self-report data may still introduce some bias; future studies could employ alternative data collection methods.

#### 4.1.2 Descriptive statistics and correlation analysis

Table 2 presents the descriptive statistics and Pearson correlation coefficients for all study variables. The data are roughly normally distributed, satisfying the requirements for parametric tests, as indicated by the skewness values being all less than 2 and the kurtosis values being less than 7. Role conflict was negatively correlated with meaning of work (*r* = −0.662, *p* < 0.001) and positively correlated with emotional exhaustion (*r* = 0.594, *p* < 0.001). A negative correlation also emerged between meaning of work and emotional exhaustion (*r* = −0.552, *p* < 0.001).

**Table 2:** Correlation analysis.

Variable	M ± SD	SK	Kur	1	2	3
Role Conflict	3.659 ± 1.205	0.139	−0.487	1		
Meaning of Work	3.956 ± 1.312	0.042	−0.863	−0.662***	1	
Emotional Exhaustion	3.567 ± 1.080	0.083	−0.096	0.594***	−0.552 ***	1

***p < 0.001, M = mean, SD = standard deviation; SK = skewness; Kur = kurtosis.

#### 4.1.3 Structural reliability and validity

Composite reliability (CR) and Cronbach’s *α* were employed to evaluate internal consistency (see Table 3). Every item factor loading was higher than the 0.70 cutoff (Hair et al., 2021). Cronbach’s α (0.866–0.903) and CR values (0.895–0.920) also exceeded the 0.70 criteria, indicating internal consistency (Leguina, 2015). The AVE for all constructs was above the 0.5 criterion, indicating convergent validity (Leguina, 2015). As shown in Table 4, all HTMT values ranged from 0.618 to 0.749, which remained under the conservative threshold of 0.85, indicating robust discriminant validity (Henseler et al., 2015). As indicated in Table 5, the square root of the AVE for each construct surpassed its inter-construct correlations, thereby satisfying the Fornell-Larcker criterion (Fornell and Larcker, 1981).

**Table 3:** Reliability and validity.

Constructs	Items	Loadings	Cronbach’s *α*	CR	AVE
RC	RC1	0.725	0.866	0.895	0.516
	RC2	0.701			
	RC3	0.720			
	RC4	0.702			
	RC5	0.703			
	RC6	0.710			
	RC7	0.749			
	RC8	0.737			
MW	MW1	0.710	0.903	0.920	0.534
	MW2	0.711			
	MW3	0.727			
	MW4	0.746			
	MW5	0.722			
	MW6	0.704			
	MW7	0.736			
	MW8	0.767			
	MW9	0.713			
	MW10	0.764			
EE	EE1	0.735	0.883	0.906	0.517
	EE2	0.719			
	EE3	0.703			
	EE4	0.715			
	EE5	0.725			
	EE6	0.727			
	EE7	0.714			
	EE8	0.731			
	EE9	0.701			

RC, Role conflict; MW, Meaning of work; EE, Emotional exhaustion.

**Table 4:** Heterotrait-Monotrait ratio (HTMT).

Variable	Meaning of work	Emotional exhaustion	Role conflict
Meaning of work			
Emotional exhaustion	0.618		
Role conflict	0.749	0.680	

**Table 5:** Fornell-Larcker criterion.

Variable	Meaning of work	Emotional exhaustion	Role conflict
Meaning of work	**0.730**		
Emotional exhaustion	−0.554	**0.719**	
Role conflict	−0.667	0.597	**0.719**

The square root of the AVE shown as the bolded diagonal values in the table.

#### 4.1.4 Confirmatory factor analysis (CFA)

To further assess model validity, we employed CFA to examine if the factor structures of the scales satisfied theoretical assumptions. Table 6 shows that the CFA results show a satisfactory model fit (Ji et al., 2024).

**Table 6:** Model fit indices.

Fit index	Reference value	Final model
χ^2^		416.091
df		321
χ^2^/df	<3	1.296
RMSEA	<0.05	0.025
GFI	>0.9	0.941
AGFI	>0.9	0.930
TLI	>0.9	0.983
CFI	>0.9	0.981
IFI	>0.9	0.983

χ^2^, Chi-square; df, degrees of freedom; RMSEA, root mean square error of approximation; GFI, goodness-of-fit index; AGFI, adjusted goodness-of-fit index; TLI, Tucker-Lewis index; CFI, comparative fit index; IFI, incremental fit index. All fit indices for the final model met the recommended reference values.

#### 4.1.5 Multicollinearity test

According to the variance inflation factor test (see Table 7), multicollinearity was not a problem, with all values remaining under 3.3 (Hair et al., 2019).

**Table 7:** Collinearity diagnostics of the structural model.

Variable	Meaning of work	Emotional exhaustion	Role conflict
Meaning of work		1.800	
Emotional exhaustion			
Role conflict	1.000	1.800	

### 4.2 Exploratory analyses: group differences by demographic variables

Results from independent-samples t-tests for binary demographic variables appear in Tables 8–10, while Tables 11, 12 contain the one-way ANOVA outcomes for age and teaching experience. The t-test results showed that role conflict differed significantly by gender and job position. Meaning of work differed significantly by job position in the t-test analyses. The ANOVA results further showed significant differences in meaning of work by age and teaching experience. According to *post hoc* LSD tests, teachers in the 20–30 and 31–40 age groups had significantly higher scores on meaning of work compared to those aged 51 and above. Furthermore, teachers having 4–10 years of experience showed significantly higher meaning of work scores than those with 1–3 years or more than 11 years of experience. No significant group differences in emotional exhaustion were found across the examined demographic variables.

**Table 8:** Independent samples *t*-test for role conflict across background variables.

Construct	Category		M ± SD	*t*	*p*
Role conflict	Gender	Male (52)	3.329 ± 1.224	−2.093	0.037
		Female (434)	3.698 ± 1.198		
	Job Position	Lead Teacher (264)	3.778 ± 1.259	2.400	0.017
		Assistant Teacher (222)	3.516 ± 1.125		
	Employment status	Permanent (268)	3.597 ± 1.184	−1.261	0.208
		Non-permanent (218)	3.735 ± 1.229		
	Kindergarten type	Public (370)	3.679 ± 1.211	0.654	0.514
		Private (116)	3.595 ± 1.191		
	Highest education	Associate Degree (431)	3.653 ± 1.214	−0.314	0.754
		Bachelor’s (55)	3.707 ± 1.142		

**Table 9:** Group differences in meaning of work by demographic variables.

Variable	Category		M ± SD	*t*	*p*
Meaning of work	Gender	Male (52)	4.240 ± 1.289	1.659	0.098
		Female (434)	3.922 ± 1.312		
	Job position	Lead teacher (264)	3.800 ± 1.328	−2.876	0.004
		assistant teacher (222)	4.141 ± 1.270		
	Employment status	Permanent (268)	3.893 ± 1.265	−1.172	0.242
		Non-permanent (218)	4.033 ± 1.366		
	Kindergarten type	Public (370)	3.909 ± 1.281	−1.399	0.162
		Private (116)	4.104 ± 1.401		
	Highest education	Associate degree (431)	3.964 ± 1.317	0.400	0.689
		Bachelor’s (55)	3.889 ± 1.277		

**Table 10:** Group differences in emotional exhaustion by demographic variables.

Construct	Category		M ± SD	*t*	*p*
Emotional exhaustion	Gender	Male (52)	3.440 ± 0.931	−0.898	0.370
		Female (434)	3.582 ± 1.096		
	Job Position	Lead teacher (264)	3.633 ± 1.151	1.486	0.138
		Assistant teacher (222)	3.489 ± 0.985		
	Employment status	Permanent (268)	3.532 ± 1.031	−0.789	0.430
		Non-permanent (218)	3.610 ± 1.137		
	Kindergarten type	Public (370)	3.611 ± 1.075	1.603	0.109
		Private (116)	3.427 ± 1.086		
	Highest education	Associate degree (431)	3.564 ± 1.081	−0.209	0.834
		Bachelor’s (55)	3.596 ± 1.075		

**Table 11:** One-way ANOVA for variables by age.

Variable	M ± SD				*F*	*p*	LSD
	a = 20–30 years (*n* = 186)	b = 31–40 years (*n* = 212)	c = 41–50 years (*n* = 57)	d = Above 51 (*n* = 31)			
Role conflict	3.747 ± 1.280	3.630 ± 1.256	3.519 ± 0.870	3.577 ± 0.881	0.673	0.569	
Meaning of work	4.065 ± 1.400	4.031 ± 1.332	3.697 ± 0.977	3.265 ± 0.849	4.363	0.005	a > d**; b > d**
Emotional exhaustion	3.686 ± 1.176	3.486 ± 1.053	3.602 ± 0.864	3.344 ± 0.965	1.612	0.186	

**p < 0.01.

**Table 12:** One-way ANOVA for variables by teaching experience.

Variable	M ± SD			*F*	*p*	LSD
	a = 1–3 years (*n* = 63)	b = 4–10 years (*n* = 178)	c = Above 11 (*n* = 245)			
Role conflict	3.935 ± 1.165	3.655 ± 1.316	3.591 ± 1.123	2.048	0.130	
Meaning of work	3.648 ± 1.375	4.207 ± 1.365	3.852 ± 1.227	5.892	0.003	b > a**; b > c**
Emotional exhaustion	3.837 ± 1.007	3.574 ± 1.184	3.493 ± 1.009	2.585	0.076	

**p < 0.01.

### 4.3 Primary analysis: mediation effect

After controlling for job position, gender, age, and teaching experience, the mediation analysis results (see Table 13) showed that role conflict was positively associated with emotional exhaustion (*β* = 0.384, *p* < 0.001) and negatively associated with meaning of work (*β* = −0.661, *p* < 0.001). Meaning of work was also negatively associated with emotional exhaustion (*β* = −0.314, *p* < 0.001). Therefore, H1, H2, and H3 were supported. It should be noted that the correlation coefficient between role conflict and meaning of work (*r* = −0.662) and the standardized regression coefficient in the mediation model (*β* = −0.661) are different statistical estimates. The former reflects the bivariate association between the two variables, whereas the latter reflects the association after controlling for gender, age, teaching experience, and job position. Their similar numerical values are coincidental.

**Table 13:** Regression results for the mediation model.

Dependent variable	Predictor variable	*β*	SE	*T*	*p*	95% CI		*R*^2^	*F*
						LLCI	ULCI		
Meaning of work	Role conflict	−0.661	0.033	−19.776	<0.001	−0.727	−0.596	0.480	88.541
	Job position	0.108	0.067	1.617	0.107	−0.023	0.240		
	Gender	−0.08	0.108	−0.077	0.939	−0.220	0.203		
	Age	−0.299	0.053	−5.626	<0.001	−0.403	−0.194		
	Teaching experience	0.160	0.065	2.465	<0.05	0.032	0.288		
Emotional exhaustion	Role conflict	0.384	0.048	7.970	<0.001	0.289	0.476	0.407	54.879
	Meaning of work	−0.314	0.049	−6.449	<0.001	−0.410	−0.219		
	Job Position	0.025	0.072	0.351	0.726	−0.116	0.166		
	Gender	−0.049	0.115	−0.426	0.671	−0.275	0.177		
	Age	−0.110	0.059	−1.881	0.061	−0.225	0.005		
	Teaching experience	−0.003	0.070	−0.049	0.961	−0.141	0.134		

The table reports the regression coefficients (β), standard errors (SE), t values, 95% confidence intervals, *R*^2^, and F values for the mediation model.

**Figure 2:**
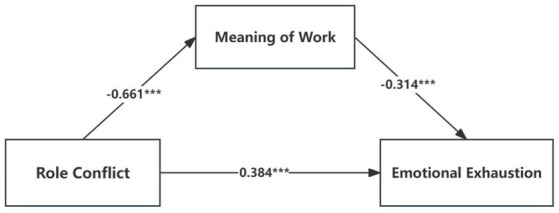
Mediation model.

As shown in Table 14, meaning of work showed a partial mediating role in the association between role conflict and emotional exhaustion (see Figure 2). The total association measured 0.591 (*95% CI* [0.518, 0.665]), with a direct association of 0.383 (*95% CI* [0.289, 0.478]), making up 64.81% of the total. The indirect association was 0.208 (*95% CI* [0.142, 0.274]), accounting for 35.19% of the total. With the 95% confidence interval for the indirect association excluding zero, H4 received support.

**Table 14:** The mediating effect of meaning of work.

Path type	*β*	Boot SE	Boot 95% CI	Proportion (%)
Total effect	0.591	0.037	[0.518, 0.665]	100.00%
Direct effect	0.383	0.048	[0.289, 0.478]	64.81%
Indirect effect	0.208	0.033	[0.142, 0.274]	35.19%

## 5 Discussion

Based on the JD-R model, this study explores the interconnections among role conflict, meaning of work, and emotional exhaustion, with a special focus on the mediating role of meaning of work. Results show a notable positive link from role conflict to emotional exhaustion, with meaning of work serving as a partial mediator. The findings of this study help us better understand preschool teachers’ professional burnout, particularly when there are various role conflicts and the distribution of work resources is directly linked to teachers’ emotional well-being.

The study’s findings, which are in line with those of Aboagye et al. (2024) in the field of early childhood education, highlight the intimate connection between role conflict as the primary cause of stress and emotional exhaustion. This pattern is not unique to China; it has also been observed internationally, such as among Indonesian preschool teachers facing dual role dilemmas (Kurniati and Nibrosurrahman, 2025). Cross-cultural research has additionally indicated that compared to preschool educators in places like Ghana and Pakistan, those in China tend to report somewhat higher levels of emotional exhaustion (Chen et al., 2023). Preschool teachers frequently have to handle the conflicting demands of administration, caregiving, and instruction, a situation that produces psychological strain and drains emotional resources, with a higher risk of emotional exhaustion as an outcome (Wang et al., 2024). In the Chinese context, it is possible that teachers face strong expectations from families, schools, and society, which could further intensify their experience of role conflict (Wu and Zhang, 2025).

This study demonstrates the mediating effect of meaning of work in the association between role conflict and emotional exhaustion, supporting the findings of Kim and Lee (2024) and Hong et al. (2025). It explains how high job expectations erode meaning of work and how the loss of this sense of purpose causes emotional exhaustion. Internationally, similar mediating mechanisms have been observed, such as among preschool teachers in Israel (Lavy, 2022). However, that study was conducted in a context in which meaning of work may be shaped more strongly by personal fulfillment and professional autonomy. In this study, meaning of work for Chinese preschool teachers may also be shaped by their broader social and professional work environment (Lei, 2024). Specifically, when teachers face conflicting demands from administration, caregiving, and teaching, their sense of meaning of work may weaken (Romero-Caraballo et al., 2024), and such a drop in meaning carries a higher risk of emotional exhaustion (Lavy, 2022). This process aligns with the JD-R model, where role conflict, acting as a job demand, consumes key psychological resources, with emotional exhaustion as a possible eventual outcome.

Regarding demographic variables, the current results do not provide robust evidence that job position is associated with emotional exhaustion. Lead teachers had a slightly higher average emotional exhaustion than assistant teachers in the descriptive data, but this difference was not statistically significant. In the adjusted mediation model, job position also did not significantly predict emotional exhaustion after controlling for gender, age, and teaching experience. Therefore, unlike some previous studies suggesting that heavier professional responsibilities, emotional labor, and administrative demands may increase burnout risk among lead teachers (Huang et al., 2026), the present study does not support a clear position-based difference in emotional exhaustion. This discrepancy may indicate that emotional exhaustion is influenced more by broader work demands and individual background characteristics than by job title alone.

Regarding age, teachers aged 20–40 reported higher meaning of work than those over 51. One possible explanation is that teachers in earlier and middle career stages may maintain stronger professional engagement, whereas older teachers may experience changes in motivation, accumulated strain, or career expectations (Zhang et al., 2025). No significant differences in role conflict or emotional exhaustion were found across age groups. Teachers with 4–10 years of experience reported higher meaning of work than those with less or more experience (Jugovic et al., 2025). This pattern may reflect the fact that novice teachers are still adapting to professional demands, whereas highly experienced teachers may experience some decline in work meaning over time (Hong et al., 2023). In addition, gender, employment status, kindergarten type, and educational attainment were not significantly associated with emotional exhaustion. Although some previous studies have reported higher emotional exhaustion among women (Firmino and Bauknecht, 2022), no gender difference was observed in the present study; however, this finding should be interpreted cautiously given the substantial gender imbalance in the sample. Overall, the present results suggest that age and teaching experience are more consistently related to meaning of work, whereas the evidence for demographic differences in emotional exhaustion is limited.

## 6 Implications and limitations

### 6.1 Theoretical implications

The relationship between role conflict, meaning of work, and early childhood teachers’ emotional exhaustion is investigated in this study using the JD-R model. This research extends the JD-R framework by showing that role conflict can diminish teachers’ meaning of work and contribute to emotional exhaustion, with meaning of work serving as a mediator. This expansion has transformed the model from a simple stimulus-reaction framework to a framework containing cognitive evaluation, highlighting the importance of maintaining meaning of work to reduce stress and professional burnout. Additionally, this study highlights how cultural influences shape meaning of work in the Chinese setting, which offers important insights for the development of intervention strategies for Chinese early childhood teachers’ emotional exhaustion.

### 6.2 Practical implications

Addressing emotional exhaustion among preschool instructors requires feasible interventions at multiple levels. At the individual level, teachers can adopt simple daily practices such as using a checklist to prioritize tasks, setting fixed time blocks for different responsibilities, keeping a brief weekly journal of three positive work experiences, and conducting a 5-min daily check-in with teaching assistants to build informal support. These actions require minimal time and no additional resources, making them readily implementable. At the kindergarten management level, concrete steps include auditing current non-teaching duties (e.g., paperwork, meeting attendance) and eliminating at least two low-value tasks per teacher per week, scheduling 10–15 min of protected break time during the workday, and establishing a rotating co-teaching system to share caregiving responsibilities. At the policy level, regional education officials can implement phased targets (e.g., reducing student-to-teacher ratio from 20:1 to 18:1 within 2 years) and reallocate existing educational budgets rather than requiring new funding. For private preschool teachers facing low employment security, a pilot subsidy program can be launched in one district and scaled up based on cost-effectiveness evaluation within 12 months.

### 6.3 Limitations and future research directions

While the present research offers important understandings of how role conflict, job meaning, and emotional exhaustion are interconnected, several limitations remain that future investigations could address. First, the cross-sectional nature of this study limits the ability to infer causal relationships. Future research could adopt a longitudinal design to track changes in teachers’ working conditions and emotional states over time, offering a deeper view of the dynamic connections among these factors. Second, the study’s sample is drawn from Chinese kindergartens in three provinces (Hainan, Hubei, Guangdong), representing southern and central regions of China. Although this regional coverage provides some diversity, it does not include all major regions of the country. Thus, caution is needed when generalizing the findings. In addition, the sample was heavily skewed toward female teachers, and this gender imbalance means that any gender-related interpretation should also be treated cautiously. Future research could include broader regional samples and more balanced demographic structures, and may also consider cross-cultural comparisons to explore differences in teachers’ mental health across cultural and educational contexts. Third, the majority of the data in this study comes from self-reporting, which could be impacted by social expectations or recall bias. Future studies can use more objective measurement techniques and more comprehensive data gathering techniques, like behavioral observation and interviews, to increase data dependability. Fourth, although we used percentile bootstrap confidence intervals (Hayes, 2022) to test the mediation effect, recent studies suggest that traditional bootstrap methods can sometimes produce overly conservative results (He et al., 2024). Future research may consider applying the adaptive bootstrap test proposed by He et al. (2024) or the causal mediation approach with variable selection developed by Jones et al. (2025) to further validate our findings. Fifth, although the discussion section offers some interpretations regarding the potential influence of Chinese social and cultural factors (e.g., strong expectations from families, schools, and society), no culture-specific variables were directly measured in this study. Consequently, these interpretations should be viewed as context-informed explanations rather than direct empirical conclusions. Future research should incorporate explicit cultural or contextual indicators—such as measures of collectivism, face culture, or societal role expectations—to examine these influences more directly. Finally, although this study explores how meaning of work plays a mediating role between role conflict and emotional exhaustion, future studies could examine more complex mediation models and consider additional potential mediators, such as psychological resilience, job resources, and social support.

## 7 Conclusion

Using the JD-R framework, this study looked into the connections between role conflict, meaning of work, and emotional exhaustion in preschool teachers. Results indicated a positive link from role conflict to emotional exhaustion and a negative link to meaning of work, with meaning of work as a partial mediator in that relationship. Nevertheless, with the indirect effect making up 35.19% of the total effect, meaning of work explains only a portion of the link between role conflict and emotional exhaustion. The remaining direct effect suggests that other mechanisms may also be involved, such as workload, organizational support, job autonomy, resilience, and other job or personal resources. Practically, these findings suggest that reducing role conflict and strengthening meaning of work through clearer role expectations, a lower non-teaching burden, and supportive school practices may help alleviate emotional exhaustion among preschool teachers.

## Data Availability

The raw data supporting the conclusions of this article will be made available by the authors, without undue reservation.
