# Supplementary material for: From stress to exhaustion: the mediating role of meaning of work in the relationship between role conflict and emotional exhaustion among preschool teachers
Source: Front Psychol. 2026 Jul 15;17:1828845. doi: 10.3389/fpsyg.2026.1828845 (PMC13415576; doi:10.3389/fpsyg.2026.1828845)
Supplement: Supplementary file 1 [file Supplementary_file_1.docx]

**Appendix A**

Description of the Mediation Analysis (PROCESS Model 4)

To test the mediating role of meaning of work in the relationship between role conflict and emotional exhaustion, this study employed Model 4 in the SPSS PROCESS macro (version 4.2) developed by Hayes (2022), which implements a basic simple mediation model using ordinary least squares (OLS) regression. In this model, role conflict was specified as the independent variable (X), meaning of work as the mediator (M), and emotional exhaustion as the dependent variable (Y). To control for potential confounding effects, gender, age, teaching experience, and job position were included as covariates in all regression equations. Specifically, the model estimates the effect of role conflict on meaning of work (path *a*), the effect of meaning of work on emotional exhaustion (path *b*), and the direct effect of role conflict on emotional exhaustion (path *c'*), with all paths controlling for the covariates. The indirect effect (a × b) was then calculated as the product of path *a* and path *b*. Given that the sampling distribution of indirect effects is often non-normal, percentile bootstrap confidence intervals were employed, and a total of 5,000 bootstrap resamples were generated to produce 95% confidence intervals (95% CIs). An indirect effect was considered statistically significant if the 95% CI did not contain zero (Hayes & Rockwood, 2017). This bootstrap procedure follows the method described in Hayes (2022), which is based on the original approach proposed by Efron (1992).

**Appendix B**

Survey Questionnaire: Role Conflict, Meaning of Work, and Emotional Exhaustion Among Preschool Teachers

*Instruction to Participants:*

This questionnaire aims to understand the relationships among preschool teachers' role conflict, meaning of work, and emotional exhaustion. All responses are anonymous, and there are no right or wrong answers. Please answer based on your actual work feelings in recent times. All data will be used solely for academic research and will be kept strictly confidential. Thank you for your support and cooperation.

*Scale Instruction:*

All items in the Role Conflict, Meaning of Work, and Emotional Exhaustion sections were rated on a 7-point Likert scale ranging from 1 (*strongly disagree*) to 7 (*strongly agree*). For each item, please select the number that best reflects your agreement.

**Demographic Information**

1. Gender: (1) Male (2) Female
2. Age: (1) 20-30 years (2) 31-40 years (3) 41-50 years (4) 51 years and above
3. Teaching experience: (1) 1-3 years (2) 4-10 years (3) More than 10 years
4. Current position: (1) Lead teacher (2) Assistant teacher
5. Employment status: (1) Permanent staff (2) Contract staff
6. Kindergarten type: (1) Public (2) Private
7. Education level: (1) Associate degree or below (2) Bachelor's degree (3) Master's degree or above

**Role Conflict**

1. At work, I often have to act in ways that are inconsistent with my personal ideas or values.
2. At work, I have to deal with things that I consider unnecessary or redundant.
3. At work, I often receive tasks but lack sufficient personnel to complete them effectively.
4. At work, I often receive tasks but lack necessary resources or materials.
5. At work, I often need to collaborate with two or more teams that have very different work styles or requirements.
6. At work, I sometimes have to bypass certain rules or policies to get tasks done.
7. At work, I receive conflicting instructions or requirements from two or more sources.
8. At work, what I do often satisfies only some people's expectations while failing to satisfy others.

**Meaning of Work**

1. At work, I have found a meaningful career.
2. At work, I view my job as contributing to my personal growth.
3. At work, my job has no impact on the world. (R)
4. At work, I understand how my job affects my life.
5. At work, I am clear about what makes my job meaningful.
6. At work, I know that my job can bring positive change to the world.
7. At work, my job helps me understand myself better.
8. At work, I have found a satisfyingly meaningful job.
9. At work, my job helps me understand the world around me.
10. At work, what I do serves a greater purpose.

*Note: (R) indicates reverse-coded item.*

**Emotional Exhaustion**

1. I feel emotionally drained from my work.
2. I feel exhausted at the end of a workday.
3. I feel fatigued when I get up in the morning and have to face another day at work.
4. Working with people all day puts a lot of stress on me.
5. I feel burned out from my work.
6. I feel frustrated by my work.
7. I feel that my work is too hard.
8. Teaching and managing children directly put a lot of pressure on me.
9. I feel I am at the end of my rope.

**Scoring**

- Role Conflict: Sum or average of items 8-15. Higher scores indicate greater role conflict.
- Meaning of Work: Sum or average of items 16-25. Higher scores indicate greater meaning of work (item 18 is reverse-coded).
- Emotional Exhaustion: Sum or average of items 26-34. Higher scores indicate greater emotional exhaustion.
